# Supplementary material for: HIVprotI: an integrated web based platform for prediction and design of HIV proteins inhibitors
Source: J Cheminform. 2018 Mar 9;10:12. doi: 10.1186/s13321-018-0266-y (PMC5845081; doi:10.1186/s13321-018-0266-y)
Supplement: Supplementary file 2 — Additional file 2. Source code of HIVProtI web server. [file 13321_2018_266_MOESM2_ESM.zip › HIVprotI_Source-code/hivprotI/weka-3-6-6/doc/weka/clusterers/OPTICS.html]

OPTICS


---


|  |  |  |
| --- | --- | --- |
| **Overview** \| **Package** \| **Class** \| **Tree** \| **Deprecated** \| **Index** \| **Help** \| **Weka's home** | |  |
| **PREV CLASS**   **NEXT CLASS** | **FRAMES**    **NO FRAMES**     **All Classes** |  |
| SUMMARY: NESTED \| FIELD \| CONSTR \| METHOD | DETAIL: FIELD \| CONSTR \| METHOD |  |


---


## weka.clusterers Class OPTICS

```
java.lang.Object
  weka.clusterers.AbstractClusterer
      weka.clusterers.OPTICS
```

**All Implemented Interfaces:**: java.io.Serializable, java.lang.Cloneable, Clusterer, CapabilitiesHandler, OptionHandler, RevisionHandler, TechnicalInformationHandler

---

``` public class OPTICS extends AbstractClusterer implements OptionHandler, TechnicalInformationHandler ```

Mihael Ankerst, Markus M. Breunig, Hans-Peter Kriegel, Joerg Sander: OPTICS: Ordering Points To Identify the Clustering Structure. In: ACM SIGMOD International Conference on Management of Data, 49-60, 1999.


BibTeX:

```
 @inproceedings{Ankerst1999,
    author = {Mihael Ankerst and Markus M. Breunig and Hans-Peter Kriegel and Joerg Sander},
    booktitle = {ACM SIGMOD International Conference on Management of Data},
    pages = {49-60},
    publisher = {ACM Press},
    title = {OPTICS: Ordering Points To Identify the Clustering Structure},
    year = {1999}
 }
```


Valid options are:

```
 -E <double>
  epsilon (default = 0.9)
```

```
 -M <int>
  minPoints (default = 6)
```

```
 -I <String>
  index (database) used for OPTICS (default = weka.clusterers.forOPTICSAndDBScan.Databases.SequentialDatabase)
```

```
 -D <String>
  distance-type (default = weka.clusterers.forOPTICSAndDBScan.DataObjects.EuclidianDataObject)
```

```
 -F
  write results to OPTICS_#TimeStamp#.TXT - File
```

```
 -no-gui
  suppress the display of the GUI after building the clusterer
```

```
 -db-output <file>
  The file to save the generated database to. If a directory
  is provided, the database doesn't get saved.
  The generated file can be viewed with the OPTICS Visualizer:
    java weka.clusterers.forOPTICSAndDBScan.OPTICS_GUI.OPTICS_Visualizer [file.ser]
  (default: .)
```

**Version:**
:   $Revision: 5538 $

**Author:**
:   Matthias Schubert (schubert@dbs.ifi.lmu.de), Zhanna Melnikova-Albrecht (melnikov@cip.ifi.lmu.de), Rainer Holzmann (holzmann@cip.ifi.lmu.de)

**See Also:**: Serialized Form

---

**Constructor Summary**

`OPTICS()`


| **Method Summary** | |
| --- | --- |
| `void` | `buildClusterer(Instances instances)`             Generate Clustering via OPTICS |
| `int` | `clusterInstance(Instance instance)`             Classifies a given instance. |
| `java.lang.String` | `database_distanceTypeTipText()`             Returns the tip text for this property |
| `java.lang.String` | `database_TypeTipText()`             Returns the tip text for this property |
| `Database` | `databaseForName(java.lang.String database_Type, Instances instances)`             Returns a new Class-Instance of the specified database |
| `java.lang.String` | `databaseOutputTipText()`             Returns the tip text for this property. |
| `DataObject` | `dataObjectForName(java.lang.String database_distanceType, Instance instance, java.lang.String key, Database database)`             Returns a new Class-Instance of the specified database |
| `java.lang.String` | `epsilonTipText()`             Returns the tip text for this property |
| `Capabilities` | `getCapabilities()`             Returns default capabilities of the clusterer. |
| `java.lang.String` | `getDatabase_distanceType()`             Returns the distance-type |
| `java.lang.String` | `getDatabase_Type()`             Returns the type of the used index (database) |
| `java.io.File` | `getDatabaseOutput()`             Returns the file to save the database to - if directory, database is not saved. |
| `double` | `getEpsilon()`             Returns the value of epsilon |
| `int` | `getMinPoints()`             Returns the value of minPoints |
| `java.lang.String[]` | `getOptions()`             Gets the current option settings for the OptionHandler. |
| `FastVector` | `getResultVector()`             Returns the resultVector |
| `java.lang.String` | `getRevision()`             Returns the revision string. |
| `SERObject` | `getSERObject()`             Returns the internal database |
| `boolean` | `getShowGUI()`             Returns the flag for showing the OPTICS visualizer GUI. |
| `TechnicalInformation` | `getTechnicalInformation()`             Returns an instance of a TechnicalInformation object, containing detailed information about the technical background of this class, e.g., paper reference or book this class is based on. |
| `boolean` | `getWriteOPTICSresults()`             Returns the flag for writing actions |
| `java.lang.String` | `globalInfo()`             Returns a string describing this DataMining-Algorithm |
| `java.util.Enumeration` | `listOptions()`             Returns an enumeration of all the available options. |
| `static void` | `main(java.lang.String[] args)`             Main Method for testing OPTICS |
| `java.lang.String` | `minPointsTipText()`             Returns the tip text for this property |
| `int` | `numberOfClusters()`             Returns the number of clusters. |
| `void` | `setDatabase_distanceType(java.lang.String database_distanceType)`             Sets a new distance-type |
| `void` | `setDatabase_Type(java.lang.String database_Type)`             Sets a new database-type |
| `void` | `setDatabaseOutput(java.io.File value)`             Sets the the file to save the generated database to. |
| `void` | `setEpsilon(double epsilon)`             Sets a new value for epsilon |
| `void` | `setMinPoints(int minPoints)`             Sets a new value for minPoints |
| `void` | `setOptions(java.lang.String[] options)`             Sets the OptionHandler's options using the given list. |
| `void` | `setShowGUI(boolean value)`             Sets the flag for displaying the GUI. |
| `void` | `setWriteOPTICSresults(boolean writeOPTICSresults)`             Sets the flag for writing actions |
| `java.lang.String` | `showGUITipText()`             Returns the tip text for this property. |
| `java.lang.String` | `toString()`             Returns a description of the clusterer |
| `java.lang.String` | `writeOPTICSresultsTipText()`             Returns the tip text for this property |

**Methods inherited from class weka.clusterers.AbstractClusterer**

`distributionForInstance, forName, makeCopies, makeCopy`

**Methods inherited from class java.lang.Object**

`equals, getClass, hashCode, notify, notifyAll, wait, wait, wait`

**Constructor Detail**

### OPTICS

```
public OPTICS()
```


**Method Detail**

### getCapabilities

```
public Capabilities getCapabilities()
```

:   Returns default capabilities of the clusterer.

    :   **Specified by:**: `getCapabilities` in interface `Clusterer` **Specified by:**: `getCapabilities` in interface `CapabilitiesHandler` **Overrides:**: `getCapabilities` in class `AbstractClusterer`
    :   **Returns:**: the capabilities of this clusterer **See Also:**: `Capabilities`

---


### buildClusterer

```
public void buildClusterer(Instances instances)
                    throws java.lang.Exception
```

:   Generate Clustering via OPTICS

    :   **Specified by:**: `buildClusterer` in interface `Clusterer` **Specified by:**: `buildClusterer` in class `AbstractClusterer`
    :   **Parameters:**: `instances` - The instances that need to be clustered **Throws:**: `java.lang.Exception` - If clustering was not successful

---


### clusterInstance

```
public int clusterInstance(Instance instance)
                    throws java.lang.Exception
```

:   Classifies a given instance.

    :   **Specified by:**: `clusterInstance` in interface `Clusterer` **Overrides:**: `clusterInstance` in class `AbstractClusterer`
    :   **Parameters:**: `instance` - The instance to be assigned to a cluster **Returns:**: int The number of the assigned cluster as an integer **Throws:**: `java.lang.Exception` - If instance could not be clustered successfully

---


### numberOfClusters

```
public int numberOfClusters()
                     throws java.lang.Exception
```

:   Returns the number of clusters.

    :   **Specified by:**: `numberOfClusters` in interface `Clusterer` **Specified by:**: `numberOfClusters` in class `AbstractClusterer`
    :   **Returns:**: int The number of clusters generated for a training dataset. **Throws:**: `java.lang.Exception` - If number of clusters could not be returned successfully

---


### listOptions

```
public java.util.Enumeration listOptions()
```

:   Returns an enumeration of all the available options.

    :   **Specified by:**: `listOptions` in interface `OptionHandler`
    :   **Returns:**: Enumeration An enumeration of all available options.

---


### setOptions

```
public void setOptions(java.lang.String[] options)
                throws java.lang.Exception
```

:   Sets the OptionHandler's options using the given list. All options
    will be set (or reset) during this call (i.e. incremental setting
    of options is not possible).


    Valid options are:

    ```
     -E <double>
      epsilon (default = 0.9)
    ```

    ```
     -M <int>
      minPoints (default = 6)
    ```

    ```
     -I <String>
      index (database) used for OPTICS (default = weka.clusterers.forOPTICSAndDBScan.Databases.SequentialDatabase)
    ```

    ```
     -D <String>
      distance-type (default = weka.clusterers.forOPTICSAndDBScan.DataObjects.EuclidianDataObject)
    ```

    ```
     -F
      write results to OPTICS_#TimeStamp#.TXT - File
    ```

    ```
     -no-gui
      suppress the display of the GUI after building the clusterer
    ```

    ```
     -db-output <file>
      The file to save the generated database to. If a directory
      is provided, the database doesn't get saved.
      The generated file can be viewed with the OPTICS Visualizer:
        java weka.clusterers.forOPTICSAndDBScan.OPTICS_GUI.OPTICS_Visualizer [file.ser]
      (default: .)
    ```

    :   **Specified by:**: `setOptions` in interface `OptionHandler`
    :   **Parameters:**: `options` - The list of options as an array of strings **Throws:**: `java.lang.Exception` - If an option is not supported

---


### getOptions

```
public java.lang.String[] getOptions()
```

:   Gets the current option settings for the OptionHandler.

    :   **Specified by:**: `getOptions` in interface `OptionHandler`
    :   **Returns:**: String[] The list of current option settings as an array of strings

---


### databaseForName

```
public Database databaseForName(java.lang.String database_Type,
                                Instances instances)
```

:   Returns a new Class-Instance of the specified database

    :   **Parameters:**: `database_Type` - String of the specified database: `instances` - Instances that were delivered from WEKA **Returns:**: Database New constructed Database

---


### dataObjectForName

```
public DataObject dataObjectForName(java.lang.String database_distanceType,
                                    Instance instance,
                                    java.lang.String key,
                                    Database database)
```

:   Returns a new Class-Instance of the specified database

    :   **Parameters:**: `database_distanceType` - String of the specified distance-type: `instance` - The original instance that needs to hold by this DataObject: `key` - Key for this DataObject: `database` - Link to the database **Returns:**: DataObject New constructed DataObject

---


### setMinPoints

```
public void setMinPoints(int minPoints)
```

:   Sets a new value for minPoints

    :   **Parameters:**: `minPoints` - MinPoints

---


### setEpsilon

```
public void setEpsilon(double epsilon)
```

:   Sets a new value for epsilon

    :   **Parameters:**: `epsilon` - Epsilon

---


### getEpsilon

```
public double getEpsilon()
```

:   Returns the value of epsilon

    :   **Returns:**: double Epsilon

---


### getMinPoints

```
public int getMinPoints()
```

:   Returns the value of minPoints

    :   **Returns:**: int MinPoints

---


### getDatabase\_distanceType

```
public java.lang.String getDatabase_distanceType()
```

:   Returns the distance-type

    :   **Returns:**: String Distance-type

---


### getDatabase\_Type

```
public java.lang.String getDatabase_Type()
```

:   Returns the type of the used index (database)

    :   **Returns:**: String Index-type

---


### setDatabase\_distanceType

```
public void setDatabase_distanceType(java.lang.String database_distanceType)
```

:   Sets a new distance-type

    :   **Parameters:**: `database_distanceType` - The new distance-type

---


### setDatabase\_Type

```
public void setDatabase_Type(java.lang.String database_Type)
```

:   Sets a new database-type

    :   **Parameters:**: `database_Type` - The new database-type

---


### getWriteOPTICSresults

```
public boolean getWriteOPTICSresults()
```

:   Returns the flag for writing actions

    :   **Returns:**: writeOPTICSresults (flag)

---


### setWriteOPTICSresults

```
public void setWriteOPTICSresults(boolean writeOPTICSresults)
```

:   Sets the flag for writing actions

    :   **Parameters:**: `writeOPTICSresults` - Results are written to a file if the flag is set

---


### getShowGUI

```
public boolean getShowGUI()
```

:   Returns the flag for showing the OPTICS visualizer GUI.

    :   **Returns:**: true if the GUI is displayed

---


### setShowGUI

```
public void setShowGUI(boolean value)
```

:   Sets the flag for displaying the GUI.

    :   **Parameters:**: `value` - if true, then the OPTICS visualizer GUI will be displayed after building the clusterer

---


### getDatabaseOutput

```
public java.io.File getDatabaseOutput()
```

:   Returns the file to save the database to - if directory, database is not
    saved.

    :   **Returns:**: the file to save the database to a directory if saving is ignored

---


### setDatabaseOutput

```
public void setDatabaseOutput(java.io.File value)
```

:   Sets the the file to save the generated database to. If a directory
    is provided, the datbase doesn't get saved.

    :   **Parameters:**: `value` - the file to save the database to or a directory if saving is to be ignored

---


### getResultVector

```
public FastVector getResultVector()
```

:   Returns the resultVector

    :   **Returns:**: resultVector

---


### epsilonTipText

```
public java.lang.String epsilonTipText()
```

:   Returns the tip text for this property

    :   **Returns:**: tip text for this property suitable for displaying in the explorer/experimenter gui

---


### minPointsTipText

```
public java.lang.String minPointsTipText()
```

:   Returns the tip text for this property

    :   **Returns:**: tip text for this property suitable for displaying in the explorer/experimenter gui

---


### database\_TypeTipText

```
public java.lang.String database_TypeTipText()
```

:   Returns the tip text for this property

    :   **Returns:**: tip text for this property suitable for displaying in the explorer/experimenter gui

---


### database\_distanceTypeTipText

```
public java.lang.String database_distanceTypeTipText()
```

:   Returns the tip text for this property

    :   **Returns:**: tip text for this property suitable for displaying in the explorer/experimenter gui

---


### writeOPTICSresultsTipText

```
public java.lang.String writeOPTICSresultsTipText()
```

:   Returns the tip text for this property

    :   **Returns:**: tip text for this property suitable for displaying in the explorer/experimenter gui

---


### showGUITipText

```
public java.lang.String showGUITipText()
```

:   Returns the tip text for this property.

    :   **Returns:**: tip text for this property suitable for displaying in the explorer/experimenter gui

---


### databaseOutputTipText

```
public java.lang.String databaseOutputTipText()
```

:   Returns the tip text for this property.

    :   **Returns:**: tip text for this property suitable for displaying in the explorer/experimenter gui

---


### globalInfo

```
public java.lang.String globalInfo()
```

:   Returns a string describing this DataMining-Algorithm

    :   **Returns:**: String Information for the gui-explorer

---


### getTechnicalInformation

```
public TechnicalInformation getTechnicalInformation()
```

:   Returns an instance of a TechnicalInformation object, containing
    detailed information about the technical background of this class,
    e.g., paper reference or book this class is based on.

    :   **Specified by:**: `getTechnicalInformation` in interface `TechnicalInformationHandler`
    :   **Returns:**: the technical information about this class

---


### getSERObject

```
public SERObject getSERObject()
```

:   Returns the internal database

    :   **Returns:**: the internal database

---


### toString

```
public java.lang.String toString()
```

:   Returns a description of the clusterer

    :   **Overrides:**: `toString` in class `java.lang.Object`
    :   **Returns:**: the clusterer as string

---


### getRevision

```
public java.lang.String getRevision()
```

:   Returns the revision string.

    :   **Specified by:**: `getRevision` in interface `RevisionHandler` **Overrides:**: `getRevision` in class `AbstractClusterer`
    :   **Returns:**: the revision

---


### main

```
public static void main(java.lang.String[] args)
```

:   Main Method for testing OPTICS

    :   **Parameters:**: `args` - Valid parameters are: 'E' epsilon (default = 0.9); 'M' minPoints (default = 6); 'I' index-type (default = weka.clusterers.forOPTICSAndDBScan.Databases.SequentialDatabase); 'D' distance-type (default = weka.clusterers.forOPTICSAndDBScan.DataObjects.EuclidianDataObject); 'F' write results to OPTICS\_#TimeStamp#.TXT - File


---


|  |  |  |
| --- | --- | --- |
| **Overview** \| **Package** \| **Class** \| **Tree** \| **Deprecated** \| **Index** \| **Help** \| **Weka's home** | |  |
| **PREV CLASS**   **NEXT CLASS** | **FRAMES**    **NO FRAMES**     **All Classes** |  |
| SUMMARY: NESTED \| FIELD \| CONSTR \| METHOD | DETAIL: FIELD \| CONSTR \| METHOD |  |


---
